# Antifungals: From Pharmacokinetics to Clinical Practice

**DOI:** 10.3390/antibiotics12050884

**Published:** 2023-05-09

**Authors:** Anália Carmo, Marilia Rocha, Patricia Pereirinha, Rui Tomé, Eulália Costa

**Affiliations:** 1 Advanced Unit for Pharmacokinetics and Personalized Therapeutics, Clinical Pathology Department, Centro Hospitalar e Universitário de Coimbra, 3004-561 Coimbra, Portugal; 2 Advanced Unit for Pharmacokinetics and Personalized Therapeutics, Pharmacy Department, Centro Hospitalar e Universitário de Coimbra, 3004-561 Coimbra, Portugalpatriciapereirinha@chuc.min-saude.pt (P.P.); 3 Clinical Pathology Department, Centro Hospitalar e Universitário de Coimbra, 3004-561 Coimbra, Portugal; rtome@chuc.min-saude.pt

**Keywords:** antifungal drugs, fungi, azole antifungals, polyene antifungals, echinocandins, pharmacogenomics, therapeutic drug monitoring, antifungal resistance

## Abstract

The use of antifungal drugs started in the 1950s with polyenes nystatin, natamycin and amphotericin B-deoxycholate (AmB). Until the present day, AmB has been considered to be a hallmark in the treatment of invasive systemic fungal infections. Nevertheless, the success and the use of AmB were associated with severe adverse effects which stimulated the development of new antifungal drugs such as azoles, pyrimidine antimetabolite, mitotic inhibitors, allylamines and echinochandins. However, all of these drugs presented one or more limitations associated with adverse reactions, administration route and more recently the development of resistance. To worsen this scenario, there has been an increase in fungal infections, especially in invasive systemic fungal infections that are particularly difficult to diagnose and treat. In 2022, the World Health Organization (WHO) published the first fungal priority pathogens list, alerting people to the increased incidence of invasive systemic fungal infections and to the associated risk of mortality/morbidity. The report also emphasized the need to rationally use existing drugs and develop new drugs. In this review, we performed an overview of the history of antifungals and their classification, mechanism of action, pharmacokinetic/pharmacodynamic (PK/PD) characteristics and clinical applications. In parallel, we also addressed the contribution of fungi biology and genetics to the development of resistance to antifungal drugs. Considering that drug effectiveness also depends on the mammalian host, we provide an overview on the roles of therapeutic drug monitoring and pharmacogenomics as means to improve the outcome, prevent/reduce antifungal toxicity and prevent the emergence of antifungal resistance. Finally, we present the new antifungals and their main characteristics.

## 1. The Fungi among Us and Their Characteristics

Fungi are eukaryotic microorganisms that may present as yeasts, molds or as a combination of both forms [1,2]. Yeasts are fungi whose growth-form is usually unicellular, reproduced via budding or fission, and they may form hyphae and pseudohyphae [3,4,5]. Molds occur in long filaments known as hyphae that may be regularly to sparsely septate with a variable number of nuclei, and grow via apical extension to form a mycelial. Fungi that have the ability to present in the form of yeast and molds are called dimorphic fungi (*Histoplasma capsulatum*, *Sporothrix schencki*). The occurrence of dimorphism is dependent on in vivo and in vitro conditions such as culture medium properties, available metabolites, incubation temperature, carbon dioxide concentration and pH of the medium, among others [1,2,5,6].

The first attempt to classify yeasts was based on physiology rather than on morphology. However, the application of sequencing methods and the use of the nomenclature principle ‘One fungus = One name’ in 2011 resulted in the identification of new species as well as changes in the genus assignment of species [1,7]. In spite of the diversity in yeast and the difficulties associated with the classification, it is considered that the medically important yeasts belong mainly to the genera *Candida*, *Cryptococcus*, *Malassezia* and *Trichosporon*.

On the other hand, the classification of filamentous fungi was based on the examination of their macroscopic and microscopic morphology, and in particular of their reproductive structures. On the basis of the types of sexual structures, fungi were organized into different phyla: Ascomycota, Zygomycota and Basidiomycota [6,8]. Nevertheless, there were a few fungi for which a sexual form was not described or could not be induced that were classified as *Deuteromycota* or *Fungi Imperfecti* [5,6,8].

However, the fungi classification was far from being understood. Sexual and asexual forms of fungi often develop independently of each other with little morphological similarities, and the morphology is dependent on the microenvironment conditions. Therefore, a microorganism may have been classified with different names and assigned different genera. Nowadays, the existence of new methods such as phylogenetic reconstruction, including phylogenomics, allows for the better organization of fungi taxonomy [1].

**Figure 1:**
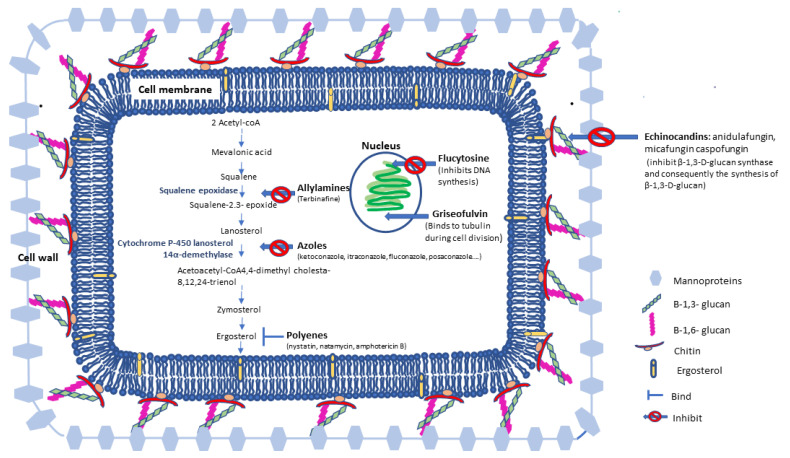
Schematic of a fungal cell describing the main characteristics of the membrane and cell wall and the sites of action of the main antifungal drugs.

Regarding the structure, fungal cells present a two-layered cell wall and a membrane with components partially different from those present in humans; see Figure 1. The major components of the cell wall are chitinous microfibrils embedded in a matrix of polysaccharides, proteins, lipids, inorganic salts and pigments that provide skeletal support and shape to the enclosed protoplast [9,10]. The major polysaccharides of the cell wall matrix are glucans such as β-1,3-D-glucan (encoded by the FKS1 and FKS2 genes), mannans (polymers of mannose), chitosan (polymers of glucosamine) and galactans (polymers of galactose). Many fungi, especially yeasts, have soluble peptidomannans as a component of their outer cell wall in a matrix of α- and β-glucans [6,8]. *Cryptococcus neoformans* produces a capsular polysaccharide composed of at least three distinct polymers: glucuronoxylomannan, galactoxylomannan and mannoprotein. Since the enzymes and several molecules involved in the fungal cell wall were different from those existing in humans, they were used as targets for the development of several antifungals. For instance, the catalytic subunit of the glucan synthase complex involved in the synthesis of β-1,3-D-glucan is the target of the antifungal drug class echinochandins.

Fungal plasma membranes have similarities to mammalian plasma membranes, but instead of having cholesterol, they have the non-polar sterol ergosterol as the main sterol; see Figure 1. Since the pathway of ergosterol biosynthesis is complex and involves different enzymes, several drugs have been developed. Allylamines such as terbinafine were developed to target the squalene epoxidase. Lanosterol 14 α-demethylase is the target of azoles. Ergosterol itself was used as a target for amphotericin B and nystatin which form a complex with the sterol affecting the membrane integrity [2,4,11]. Since enzymes, glucans and ergosterol are specific types of fungi, it was expected that these antifungal drugs would be effective in the treatment of fungi-associated infections. However, some drawbacks are associated with similarities between fungal and mammalians cells that contribute to the development of adverse reactions; an increase in the number of patients with a compromised immune system unable to act upon a fungi infection, alterations in the fungi epidemiology and the development of fungi resistance to the antifungal have been limiting the efficacy of the available drugs.

In fact, pathogenic and opportunistic fungi are an increasing health problem. The most common yeast isolated in patients’ samples is *Candida* sp., while *Aspergillus* sp. is the most commonly isolated filamentous fungus. Other fungi such as *Fusarium* sp., *Scedosporium* sp., *Penicillium* sp. and *Zygomycetes* are now being more frequently identified and are considered to be life-threatening species [12,13].

The incidence of candidemia varies between 3.5 and 16.5/1000 admissions, depending on studies and countries with a high mortality of up to 60% in critically ill patients [14,15,16]. Epidemiological studies have pointed out that most of these infections are healthcare-associated mainly due to the use of immunosuppressive therapies and invasive procedures [14,17,18]. The delay in starting antifungal therapy has a major impact on the mortality rate: mortality may vary from 10% if antifungals are initiated in the 12 h following the first positive blood culture sample versus >30% if treatment is delayed for more than 48 h [17,19,20,21].

The epidemiology of yeast-associated infections also evidences an increase in the incidence of non-*Candida albicans* species that present a more resistant profile, highlighting the need for well-defined diagnostic and treatment strategies [15,22,23]. The emergence of *Candida auris* is one of the most worrying issues, having been considered by CDC, in 2019, as one of the five “urgent threats” [24]. *Candida auris* was first identified in 2009 in Japan, and since then it has been identified in many outbreaks worldwide. The antifungal profile of *Candida auris* varies from very resistant to fluconazole, to variably resistant to amphotericin B, and to acquiring resistance to echinocandins [24,25]. Moreover, *Candida auris* isolates resistant to the three major classes of antifungal agents have been identified in the US and other countries, raising concern regarding healthcare transmission [15,17,22,25,26].

Regarding filamentous fungi, the occurrence of invasive aspergillosis is one of the major concerns being estimated to cause more than 200,000 life-threatening infections each year [18,27,28]. Mortality rates range from 30% to 90%, varying by patient population as well as by severity and duration of immunosuppression [29,30,31,32]. As with the *Candida* species, there is a serious concern regarding the emergence of resistance mainly associated with the treatment of *Aspergillus* species [29,30,31,33,34].

Overall, it is estimated that there is a total of 1.2 billion people suffering from fungal infection. The large majority of situations are easily treated but, as previously described, there is growing concern regarding the alteration in the epidemiology and the emergence of resistance to antifungal drugs [16,35]. Establishing an adequate therapeutic plan is crucial to ensure correct identification of the fungi and understand the mechanism of action of the antifungals as well as the pharmacokinetic (PK) and pharmacodynamic (PD) characteristics of these drugs. Moreover, it is mandatory to investigate the metabolism and signaling pathways that differentiate fungal cells from mammalian cells in order to develop more effective drugs with fewer adverse effects.

## 2. Antifungal Drugs and Clinical Use

**Figure 2:**
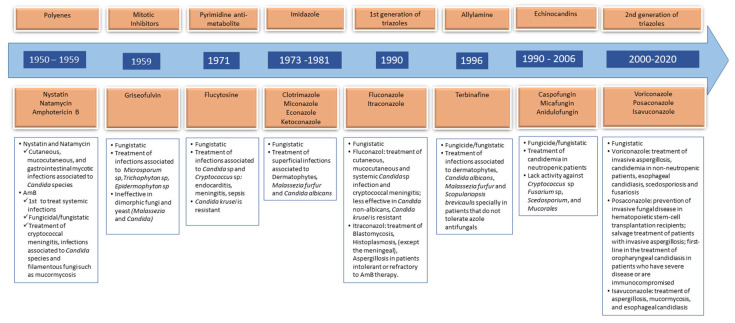
Timeline of antifungal drugs and main clinical indications.

Antifungal drugs can be divided into six major groups: azoles, echinocandins, polyenes, pyrimidine analogs, allylamines and mitotic inhibitors; see Figure 2 and Table 1 [31,36,37,38]. In the next sections, the main properties of the antifungals and their clinical use will be discussed. The authors also focus on the need to use pharmacogenomic analysis and therapeutic drug monitoring as tools to help in the establishment of more adequate therapeutic plans; see Table 2 and Table 3.

### 2.1. Polyene Antifungals

Polyenes originally extracted from *Streptomyces nodosus* bind irreversibly to the ergosterol of the fungal membrane. This binding induces the formation of ion channels and the loss of protons and monovalent cations, resulting in depolarization and concentration-dependent cell killing. Polyenes also produce oxidative damage due to the formation of free radicals that subsequently increase membrane permeability [39,44,45,47]. One of the first known antifungals was polyene nystatin that was discovered in 1949 and patented in 1957 by Elizabeth Lee Hazen and Rachel Fuller Brown [38,46]. Severe adverse effects associated with the parenteral use of nystatin and difficulties associated with its solubilization limited the development of a better formulation. Since then, nystatin has mainly been used to treat cutaneous, mucocutaneous and gastrointestinal mycotic infections associated with the *Candida* species. Natamycin is also a polyene, discovered in 1955, that, like nystatin, is only used topically, especially in the treatment of keratitis. Therefore, it is considered that the use of antifungals to treat systemic antifungal infections started in 1958 with the introduction of amphotericin B-deoxycholate by Squibb Laboratories [45].

Amphotericin B (AmB)-deoxycholate is a polyene developed from more than 200 polyene macrolide antibiotics [39,44,45]. Nevertheless, despite the success obtained in the treatment of fungal infections, the use of amphotericin was associated with serious adverse effects such as infusion-related toxicity and nephrotoxicity. The toxicity was associated with the non-selective action of AmB, that in spite of having over ten-fold higher affinity for fungal ergosterol also has affinity for mammalian cholesterol due to its similarity with ergosterol. In addition, the infusion-related toxicity (fever and nausea) is due to the inflammatory signaling pathway initiated by the mammalian cells (Toll-like receptor 2 and CD14) in response to the microbial origin of AmB. The search for a new formulation led, in the late 1970s, to liposomal drug encapsulation which reduced but did not eliminate the nephrotoxicity [43,48,49]. AmB is usually administered intravenously since it is poorly absorbed from the gastrointestinal tract, is widely distributed to all tissues except to the central nervous system and elimination is mainly via slow hepatic metabolism with a small fraction of the drug excreted in the urine [39,45,50]. The drug exhibits concentration-dependent fungicidal activity with Cmax/MIC (minimum inhibitory concentration) and the PD index being the most predictive of efficacy; see Table 2 [44,45,48,51]. However, the benefits of dosage escalation may not be worth it, since the risk of nephrotoxicity and severe hypokalemia will significantly rise. Moreover, AmB maintains fungicidal activity and fungal growth inhibition after the concentration has fallen below the MIC of the infecting organism [44,45,52]. Liposomal AmB is the main antifungal drug used in invasive fungal infection associated with *Histoplasma capsulatum*, *Coccidioides immitis*, *Candida* species, *Blastomyces dermatitidis*, *Rhodotorula*, *Cryptococcus neoformans*, *Sporothrix schenckii*, *Mucor* and *Aspergillus fumigatus* in critically ill patients [48,50,53]. In spite of the large spectrum of activity, *Aspergillus terreus*, *Pseudallescheria boydii* and *Fusarium* sp. are often resistant to the drug [48,49,54,55].

### 2.2. Flucytosine

In the 1950s, it was discovered that flucytosine (5-fluorocytosine, [5-FC]), a synthetic fluorinated pyrimidine analog that lacked antineoplastic activity, exhibited antifungal activity [43,54,55]. Flucytosine is available via oral and intravenous formulation and once inside the fungus, it is converted by cytosine deaminase to the active form 5-fluorouracil (5-FU) and to fluoro-deoxyuridylic. 5-FU is incorporated into the RNA strand by competing with uracil and disrupts RNA synthesis, and fluoro-deoxyuridylic inhibits DNA synthesis [43]. The production of 5-FU makes flucytosin highly toxic since this metabolite inhibits the thymidylate synthase of the host cells and consequently inhibits the production of deoxythymidine mono-phosphate, essential for DNA replication and repair [43,56].

Flucytosine has a concentration-independent PD profile and a narrow therapeutic index with toxicity associated with peak levels; see Table 2. Its use is recommended to treat infections caused by the *Candida* species and *Cryptococcus neoformans*. However, *Candida krusei* exhibits decreased susceptibility to flucytosine [43,47,51,57]. Due to the adverse effects (bone marrow depression, leukopenia, thrombocytopenia, hepatitis, diarrhea, nausea and vomiting), its use in monotherapy is reduced, but combined therapeutic schemes with AmpB or azoles have been successfully established to treat multidrug-resistant emerging fungi such as *Cryptococcus neoformans* and *Candida auris*. It is considered that flucytosine alone has fungistatic activity, but combined with amphotericin B, it has a fungicidal effect [19,26,56,57].

### 2.3. Griseofulvin

In 1958, Gentle J.C. evidenced that griseofulvin, a metabolic product of *Penicillium griseofulvum*, discovered in 1939, had an effect on dermatophytosis [58]. This discovery led to the development of a successful oral formulation which has been used until today for the treatment of tinea capitis and other forms of dermatophytosis and onychomycosis. Griseofulvin binds to tubulin and inhibits the assembly of the microtubules and the formation of the mitotic spindle, acting as a fungistatic drug against the *Trichophyton*, *Microsporum* and *Epidermophyton* species. However, it is ineffective in treating infections associated with dimorphic fungi, yeast (*Malassezia* and *Candida*) or chromoblastomycosis [42,59,60]. Griseofulvin is absorbed from the gastrointestinal tract and reaches the keratinized structures of the skin, justifying its success in treating dermatophytosis and onychomycosis. The biological half-life of griseofulvin is 9 to 24 h in the blood and it is excreted in urine and feces after being metabolized by the liver microsomal enzyme system [42]. As for adverse effects, nausea, vomiting and diarrhea are the most frequently reported, but photosensitivity, petechiae, pruritus and urticaria have also been reported. In addition, griseofulvin should not be used in patients with porphyria, as it decreases the bioavailability of warfarin, resulting in a decreased anticoagulant effect, and it also causes disulfiram-like reactions with ethanol. Considering the adverse effects and the hazardous drug–drug interactions, the use of griseofulvin is in most cases replaced by terbinafine and azoles [42,60].

### 2.4. Imidazoles

In the 1950s, interest was raised in the azole compounds that act by inhibiting lanosterol 14α-demethylase (a CYP450-dependent enzyme encoded by the ERG11 gene) and blocking ergosterol synthesis. The first azole presenting antifungal activity was the topical imidazole chlormidazole. Later, three new imidazole compounds were developed: clotrimazole, miconazole and econazole [31,37,61,62]. Clotrimazole was the first oral azole and exhibited excellent activity against yeasts and dermatophytes. However, it was realized that the PK profile was difficult to adjust since it induced liver microsomal enzymes involved in its metabolism, diminishing its antifungal activity. In addition, it had serious adverse effects (gastrointestinal, hepatic and adrenal dysfunction) that limited its systemic use [37,62]. Since then, clotrimazole has only been used in topical formulations to treat infections associated with dermatophytes and the *Candida* species such as tinea versicolor, oral thrush and vaginal candidiasis [31,37,61].

Miconazole was the first imidazole available for intravenous administration, but due to adverse reactions, it is no longer available [31,37,63].

Econazole is an imidazole only available for topical administration to treat superficial mycoses such as tinea versicolor and cutaneous candidiasis [31,37,61,64].

In 1981, the Food and Drug Administration (FDA) approved the use of another imidazole, ketoconazole. For a few years, it was used to treat chronic mucocutaneous candidiasis, blastomycosis, histoplasmosis and paracoccidioidomycosis [31,37]. However, the results from several studies evidenced that ketoconazole had some drawbacks: the absorption was dependent on gastric pH (high pH decreased absorption) and it had poor penetration in the blood–brain barrier, limiting its use in treating fungal meningitis. In addition, serious adverse effects were reported such as severe hepatotoxicity and adrenal insufficiency due to its role in the inhibition of enzymes from the steroid synthesis pathway and clinically important drug interactions [31,65,66,67]. In 2013, the review of the available data on the efficacy and safety of ketoconazole containing medicines for oral use by the European Medicines Agency’s Committee on Medicinal Products for Human Use (CHMP) recommended the suspension of the marketing authorizations of oral ketoconazole-containing medicines throughout the European Union (EU). The CHMP concluded that the risk of liver injury was greater than the benefits of treating fungal infections [68]. Currently, ketoconazole is available for topical use, to treat infections such as athlete’s foot, jock itch, tinea corporis and pityriasis (tinea) versicolor.

Other imidazoles were developed and commercialized for topical use to treat dermatomycoses (*Trichophyton* sp., *Epidermophyton floccosum* and *Microsporum* sp.), tinea versicolor (*Malassezia furfur*) and cutaneous and vaginal candidiasis: bifonazole, butoconazole, fenticonazole, isoconazole, oxiconazole, sulconazole and tioconazole [37].

### 2.5. Triazole Compounds

To overcome the difficulties associated with imidazoles, in 1990, fluconazole was developed, the triazol that can be administrated intravenously and orally, with good penetration in the blood–brain barrier (CSF levels of almost 80% of the corresponding serum levels, independently of dose) [69,70]. The PK profile is not dependent on gastric pH; it presents complete absorption after oral administration and has a serum half-life that allows for once-daily dosing; see Table 2. It presents renal clearance, with 70–80% of the unchanged drug excreted in the urine, requiring dose adjustment in patients with renal failure; see Table 2 [47,71]. Fluconazole was approved for the treatment of oropharyngeal, esophageal, vaginal, peritoneal and genito-urinary candida infections, disseminated candidiasis and cryptococcal meningitis. The drug also has good activity against coccidioidomycosis and is a good alternative to ketoconazole in chronic mucocutaneous candidiasis; however, it is nevertheless considered that *Candida dubliniensis*, *Candida guilliermondii*, *Candida kefyr*, *Candida lusitaniae* and *Candida krusei* are resistant to fluconazole [72].

Due to the increasing number of infections associated with *Candida non-albicans* and the emergence of filamentous fungi such as *Trichosporon* sp., *Fusarium* sp., *Scedosporium prolificans*, *Mucoraceae* and *Dematiaceous* presenting reduced susceptibility to fluconazole, new azoles were developed [71,73].

Itraconazole, that became available two years after fluconazole, presented a better spectrum of activity against *Candida* sp., *Aspergillus* sp., *Cryptococcus neoformans*, *Coccidioides immitis*, *Histoplasma capsulatum*, *Blastomyces dermatitidis*, *Paracoccidioides brasiliensis*, *Sporothrix schenckii* and some *Phaeohyphomycetes* [44]. However, it has poor penetration in CSF (concentrations in CSF are negligible), the absorption upon oral absorption is dependent on the gastric pH and it has adverse effects that limited its effectiveness and use; see Table 2 [70]. The comparison of the toxicity profile evidenced that itraconazole had a better toxicity profile than ketoconazole but a less favorable profile than fluconazole. Itraconazole is excreted in urine (35%) and in feces [71,73]. Itraconazole is not recommended to treat cryptococcal meningitis but it may be used as a prophylaxis against cryptococcal meningitis, particularly in patients with CD4 counts <100 cells/μL [74].

Despite the improvements in the PK and pPD profiles of the first generation of triazoles, they presented several limitations, such as the spectrum of activity, the development of resistance, the induction of hazardous drug–drug interactions and toxicity. To overcome these limitations, a second generation of triazoles was developed, which includes voriconazole (2002), posaconazole (2006) and isavuconazole (2015) [73].

Voriconazole is a derivative of fluconazole; it is available for oral and intravenous administration, and has similar PK properties. It has an oral bioavailability >95% and takes 1 to 2 h to reach maximum concentrations; see Table 2 [20,75]. However, it is also associated with acute toxicities (neurotoxicity, prolonged QT interval and liver function test abnormalities) and long-term toxicities (photosensitivity, fluorosis and periostitis) [20,75]. In May 2002, the Food and Drug Administration recommended the use of voriconazole for the treatment of invasive aspergillosis and refractory infections of *Scedosporium apiospermum* and *Fusarium* sp. [20,75,76]. Guidelines from the Infectious Diseases Society of America (IDSA) also recommended voriconazole as a primary therapy for invasive aspergillosis and as an alternative therapy for candidemia [21,32]. However, in recent years, *Aspergillus fumigatus*, one of the main entities associated with invasive aspergillosis, developed mechanisms that showed high resistance to voriconazole and were associated with treatment failure [34,77,78,79,80].

Posaconazole, derived from itraconazole, has a broader spectrum of action, and is used to treat infections associated with *Aspergillus* and *Fusarium* species and *Mucorales*. It is available for oral and intravenous administration, but when administered orally it may not achieve sufficient concentrations in the bloodstream to treat hematogenous infection [76]. It has an elimination half-life of approximately 20 h, it is not metabolized through the cytochrome P450 enzyme system and it is excreted unchanged in feces; see Table 2 [54,81]. As with the previous azoles, its use is associated with gastrointestinal disturbances, headaches and elevated hepatic transaminases [31,37,54,73].

Posaconazole use is recommended for the prevention of invasive fungal disease in hematopoietic stem-cell transplantation (HSCT) recipients with graft-versus-host disease, and in patients with acute myelogenous leukemia or myelodysplastic syndromes who undergo intensive chemotherapy. It is also approved for the salvage treatment of patients with invasive aspergillosis, and to treat oropharyngeal candidiasis as a first-line therapy in patients who have severe disease or are immunocompromised [81,82,83].

The new triazole, isavuconazole, is the active metabolite of the prodrug isavuconazonium sulfate, a water-soluble prodrug cleaved and almost entirely cleared by plasma esterase [52]. Isavuconazole, the active moiety, has an elimination half-life of approximately 56–130 h once absorbed, and does not reach steady state until Day 14 with once-a-day dosing; see Table 2. It is available for oral and intravenous treatment. This new drug seems to have a spectrum of activity similar to voriconazole and posaconazole but with less interactions and with reduced nephrotoxicity (allowing its use in chronic renal failure), hepatotoxicity, visual effects and neurotoxicity in direct comparison with voriconazole. In 2016, the randomized controlled SECURE trial found isavuconazole to be non-inferior to voriconazole for the treatment of invasive aspergillosis and mucormycosis [52,54].

### 2.6. Terbinafine

Terbinafine is a synthetic allylamine, that has been available since the early 1990s, that acts as a fungicide drug. The drug inhibits squalene epoxidase, causing the accumulation of squalene and interfering with ergosterol synthesis. It is available for oral and topical use. Once taken orally, terbinafine concentrates in the skin and nail beds and has relatively low bloodstream concentrations, which is what makes it a good drug to treat onychomycosis and cutaneous fungal infections [84]. It is used to treat infections associated with dermatophytes (*Trichophyton*, *Microsporum* and *Epidermophyton*), *Candida albicans* and *Scopulariopsis brevicaulis*, especially in patients that do not tolerate azole antifungals [40,76,84]. A systematic review comparing efficacy and safety of systemic antifungals for tinea capitis in children found that both griseofulvin and terbinafine were effective, but terbinafine was more effective against *Trichophyton tonsurans* and griseofulvin was more effective against *Microsporum canis*[85].

### 2.7. Echinocandins

Another group of antifungal drugs is the lipopeptides echinocandins that act by competitively inhibiting β-1,3-D-glucan synthase, essential for the synthesis of β-1,3 glucan, a component of the cell wall of fungi that is not present in mammalian cells [36,41,86]. According to the expression of glucan synthase and the polymerization of β-1,3-D-glucan, echinocandins may have fungicidal activity such as in the *Candida* species or fungistatic activity such as against the *Aspergillus* species [47,76]. Its development started in 1974 with echinocandin B, which had good activity against fungi but had hemolytic effects [36,41,46,87]. It was replaced by cilofungin, which had good activity against fungi and a reduced hemolytic effect, but had difficulties with the solvent being toxic, determining the suspension of the clinical trial in 1988 [36,41,87]. In 1985, two pneumocandins were isolated from *Glarea lozoyensis*, from which pneumocandin B0 was used to synthesize in 1992, the caspofungin acetate approved as a drug for the prevention of fungal infections in adult patients by the U.S. Food and Drug Administration (FDA) in January 2001 [87]. Since then, other echinocandins have been obtained: micafungin was discovered in Japan and approved in 2005, which has reduced hemolytic activity [88]; anidulafungin resulted from an improvement in echinocandin B and was approved in 2006 for the treatment of esophageal candidiasis, candidemia and deep tissue candidiasis [89]; and rezafungin (CD101) is a next-generation echinocandin in phase 3 of a clinical trial, developed from anidulafungin. Unlike the previous echinocandins that were administered once a day intravenously, this one has a longer half-life which allows for weekly administration [90]. Echinocandins are used intravenously, with good distribution in the tissues, and are eliminated largely via hepatic metabolism; see Table 2. These drugs present concentration-dependent activity and are considered by the Infectious Diseases Society of America and European Society for Clinical Microbiology Diseases to be the first-line drugs for the treatment of invasive *Candida* infection. However, in recent years, there have been several reports of resistance to echinocandins and clinical failure related to the treatment of *Candida glabrata*, *Candida parapsilosis* and *Candida guillermondi* [91,92]. Moreover, they lack activity against the *Cryptococcus* species, *Fusarium*, *Scedosporium* and *Mucorales*, that often develop in severely immunocompromised patients [72,76,90,93].

## 3. Pharmacogenomics and Therapeutic Drug Monitoring

Pharmacogenomics is defined as the study of the impact of genetic variation on the PK and PD profiles of the drugs [94,95].

Therapeutic drug monitoring (TDM) is considered to be a key point in therapeutics, since in many patients the correlation between guideline-based antifungal dosage and serum drug concentrations is poor, with many patients being outside the therapeutic target [96,97].

Therefore, the identification of gene variants allied to TDM reduce the likelihood of adverse drug reactions and optimize therapeutic efficacy. Together, pharmacogenomics and TDM allow for a change in the paradigm from ‘one-size-fits-all’ prescription to a “personalized” precision prescription [94,96].

The identification of gene variants is dependent on the use of genotyping methods but the interpretation and valuation of the results is based on peer-reviewed evidence-based guidelines. One of the sources of these guidelines is the Clinical Pharmacogenetics Implementation Consortium (CPIC) that is based on the Pharmacogenomics Knowledgebase (PharmGKB), an online resource created by the National Institute of Health [95].

Regarding TDM, there are several methods that can be used to measure the serum concentrations of antifungal drugs, such as liquid chromatography with ultraviolet detection (HPLC–UV), liquid chromatography–mass spectrometry (LC-MS) and surface-enhanced Raman spectroscopy combining chemometrics [98,99,100]. Each technique has advantages and disadvantages, but the LC-MS is in an expanding mode among analytical quantification methods, due to its high sensitivity and specificity. Considering that most of these analytical methods are “in house”-developed and lack standardization, despite some guidelines on method development, the incorporation of an external quality assurance program is a fundamental tool for method validation, clinical utility assurance, standardization and safety [101,102,103].

In the next section, a brief review on the pharmacogenomic characteristics and the need for TDM will be described for each antifungal class.

### 3.1. Polyenes

According to the CPIC, there are no variant or clinical annotations regarding polyenes. In addition, there is no evidence to perform TDM in patients treated with nystatin or AmB. However, due to the adverse effects associated with the use of AmB, all patients receiving any formulations of amphotericin B should have their renal and hepatic function, electrolytes (particularly potassium and magnesium) and complete blood count monitored [96,104].

### 3.2. Azoles

Patients treated with imidazole antifungals do not need to undergo pharmacogenomic evaluation and there is no current indication for TDM. However, due to adverse events, in the long-term treatment of ketoconazole, it is recommended that liver function at baseline and during therapy be evaluated, with hepatic enzymes being checked weekly. In addition, patients presenting a risk of adrenal insufficiency should have their adrenal function evaluated [65,67,68,105].

Regarding triazole antifungals, pharmacogenomic evaluation is recommended for voriconazole and posaconazole. PharmaGKB presents 3 prescribing info, 6 drug label annotations and 4 clinical annotations. The CPIC published a guideline for voriconazole and the European Medicines Agency (EMA) also published clinical annotations for voriconazole and posaconazole [20,81,83,106].

Voriconazole is metabolized in vitro predominantly by CYP2C19, with contributions from CYP3A and CYP2C9. In addition, it is an inhibitor of CYP3A4, CYP2C19 and CYP2C9. It is considered that the interpatient variability observed in voriconazole concentrations is associated not only with age, hepatic function and drug interaction but also with the variant CYP2C19 alleles. The normal function of the CYP2C19 enzyme corresponds to the presence of the wild-type CYP2C19*1 allele, the absence of function is associated with the presence of the *2 (c.681G>A;rs4244285) allele and the increased function of the enzyme is associated with the CYP2C19*17 allele (c.-806C>T; rs12248560). There are other CYP2C19 alleles associated with a decreased function, but they are rare with the exception of CYP2C19*3 (c.636G>A; rs4986893) in Asian people. According to CPIC recommendations, a patient presenting CYP2C19 (*1/*1) is considered to be a normal metabolizer and clinicians may initiate therapy with the recommended standard of care dosing of voriconazole (strong recommendation). Patients presenting CYP2C19 (*2/*2, *2/*3 or *3/*3) are considered to be poor metabolizers and may need dose-adjusted trough concentrations of voriconazole. A patient presenting CYP2C19 (*17/*17) is seen as an ultrarapid metabolizer, and the probability of achieving the therapeutic concentrations is reduced. Both cases, the use of an antifungal that is not dependent on CYP2C19 metabolism is recommended (moderate recommendation), such as isavuconazole, liposomal amphotericin B or posaconazole [94,95,96].

Posaconazole is not metabolized to a significant extent via the CYP enzyme system; however, it is a potent inhibitor of CYP3A4, which justifies the clinical annotation of EMA. Therefore, plasma concentrations of drugs that are metabolized by CYP3A4 (tacrolimus, sirolimus, atazanavir, HMG-CoA reductase inhibitors (atorvastatin, lovastatin and simvastatin) and benzodiazepine, among others) may be increased by posaconazole [83,106].

Regarding TDM of triazole antifungals, the recommendations vary according to the PK/PD profile. It is not routinely required for fluconazole [107]. However, it may be indicated in rare circumstances such as in CNS disease, unstable patients receiving renal supportive care or the treatment of an organism with a high MIC [74,107].

Nevertheless, for patients on itraconazole, TDM is routinely recommended because there is clinical evidence of drug exposure–toxicity. There have also been reports of treatment failure due to a sub-optimal concentration and there are patient characteristics that make the evaluation of the PK profile difficult. Considering the report from the British Society for Medical Mycology, itraconazole TDM should be performed to minimize drug-related toxicity. The first determination should only be carried out at the end of the first week and then at regular intervals that are appropriate to the clinical context; a trough concentration of 0.5–1 mg/L should be the target for the prevention and treatment of invasive fungal infections [97,107,108].

For patients on voriconazole, TDM is also recommended. In fact, the evidence of drug exposure–toxicity relationships together with the existence of gene polymorphisms, drug to drug interactions, altered gastrointestinal absorption and body weight makes it difficult to adjust the dose of voriconazole. This recommendation was supported by a randomized controlled trial evidencing that the outcomes (complete or partial response) in patients undergoing TDM were significantly better than those in the non-TDM group [109,110]. According to the report from the British Society for Medical Mycology, the following are recommended: the initial sampling should occur in the first 2–5 days of therapy and then regularly; a lower target with a trough concentration of >1 mg/L or a trough with MIC ratio of 2–5 should be achieved whenever there is an established disease; and to minimize drug-related toxicity, the trough concentration should be <4–6 mg/L [108]. In addition to serum drug monitoring, it is also important to evaluate liver and kidney function, electrolytes (including magnesium and calcium) and lipase, if a patient has a risk of pancreatitis, and an ophthalmic exam is necessary for patients receiving voriconazole for greater than 28 days due to the possible occurrence of toxic optic neuropathy [105,111].

For reasons similar to those described for voriconazole, patients treated with posaconazole should also have TDM performed. The first determination should only be carried out at the end of the first week since a steady-state trough concentration is not apparent until then, and with changes to dosage it will take a further 7 days before a new steady state can be established. Moreover, the lower target concentration recommended for patients receiving posaconazole for prophylaxis is a trough concentration of >0.7 mg/L, and for patients with established infection the lower target recommended is a trough concentration of >1.0 mg/L [97,107,108]. In addition to drug concentration monitoring, liver and kidney function and electrolytes (including magnesium and calcium) should also be determined at baseline and during treatment [105].

Regarding isavuconazole, the results from the SECURE study did not find a relationship between exposure–response and concluded that TDM is not recommended. However, considering that it is a triazol, in a prolonged treatment it is desirable to evaluate liver function periodically [107,112].

### 3.3. Terbinafine

There is no recommendation regarding terbinafine metabolism and gene variants. However, there is an annotation of the FDA Label for terbinafine and drug interactions. Terbinafine is an inhibitor of the CYP2D6 isozyme and may convert extensive CYP2D6 metabolizers into poor metabolizers. Therefore, the coadministration of terbinafine and of drugs metabolized by CYP2D6 (tricyclic antidepressants, selective serotonin reuptake inhibitors, beta-blockers, antiarrhythmics class 1C and monoamine oxidase inhibitors Type B) should be accompanied by a reduction in their doses. Terbinafine shows no supporting evidence to suggest that TDM is necessary for its utilization in prophylaxis, treatment or toxicity [105].

### 3.4. Echinocandins

For patients undergoing echinocandin therapy, considering the reports of fungi resistance, clinical failures and the hypothesis that suboptimal concentration may potentiate the occurrence of resistance, TDM seems to be necessary. In fact, in a recent review, Kim et al. proposed TDM for echinocandins based on the exposure–response relationship, PK/PD markers and factors affecting PK in order to prevent suboptimal drug exposure, maximize efficacy and prevent acquired drug resistance. The authors also suggest that TDM is particularly important in critically ill patients, in obese patients and in pediatric patients [91,113].

### 3.5. Griseofulvin

For griseofulvin, there is no recommendation to perform pharmacogenomic studies or TDM. Moreover, routine laboratory testing appears to be unnecessary in adults and children on griseofulvin, without underlying hepatic or hematologic conditions [42,59,114].

### 3.6. Flucytosine

Considering flucytosine, PharmGKB has one piece of prescribing information and one drug label annotation based on the Dutch Pharmacogenetics Working Group Guideline. Flucytosine is almost exclusively excreted unchanged in the urine but a small part is deaminated to 5FU. The enzyme dihydropyrimidine dehydrogenase (DPD) is encoded by the DPYD gene and converts 5FU into inactive metabolites. Genetic variations in the DPYD can lead to a DPD enzyme with reduced or absent activity. The most relevant polymorphism is DPYD*2A (rs3918290). This variant results in a truncated protein that is functionally inactive, and when in homozygosity it is recommended to replace flucytosine with an alternative drug. There are other variants such as DPYD*13A (rs55886062) and HapB3 (rs7501718) that are associated with decreased DPD activity being recommended to reduce the starting dose. The increase in 5FU is associated with neutropenia, thrombocytopenia, hepatitis and diarrhea, which justifies the implementation of TDM. Drug monitoring should be performed in the majority of patients receiving flucytosine and the first serum determination may be conducted 72 h after the first administration and then regularly after. To minimize flucytosine-drug-related toxicity, the peak concentration should vary between 30 and 80 mg/L since a serum concentration >100 mg/L was associated with myelotoxicity. In addition to drug concentration monitoring, liver and kidney function and full blood count should also be evaluated at baseline and during treatment. [43,107,108]

Table 3 summarizes the recommendations for TDM and pharmacogenomic evaluations according to the available guidelines and recommendations.

## 4. Development of Resistance to Antifungals

A resistant strain can be defined as as a strain that has a minimal inhibitory concentration (MIC) for a particular antifungal above the specific clinical breakpoint. Resistance to antifungals may be intrinsic, due to ineffective binding of the drugs and/or to increased antifungal extrusion, such as in some strains of *Aspergillus* sp., *Candida krusei* and most *Candida auris* isolates that are intrinsically resistant to fluconazole [115,116,117,118]. However, resistance may also be acquired, which is an emerging problem in the treatment of fungal diseases.

Recent evidence has demonstrated that the percentage of resistant fungi has grown in recent years. It has been hypothesized that the widespread use of antifungal drugs, the use of a suboptimal concentration for a prolonged period of time and the use of fungicides in agriculture contribute to the occurrence of genetic alterations that turn fungi resistant to the drugs. The fungi most frequently associated with antifungal resistance are *Candida* sp., in particular non-*Candida albicans* sp. and *Aspergillus* sp. [22,115,119].

The evaluation of antifungal resistance is based on several laboratory methods such as broth microdilution (the gold standard for antifungal susceptibility testing), Etest and disk diffusion. Broth microdilution was recommended by the Clinical Laboratory Standards Institute (CLSI) and the European Committee on Antimicrobial Susceptibility Testing (EUCAST) and allows one to evaluate the susceptibility of fungi and determine clinical ‘break points’ for the antifungals [117,120].

However, there are several drawbacks associated with the use of these methods. First of all, it is necessary to obtain a culture of the fungus and to perform its correct identification, which may take several days. Then, the execution of these methods is time-consuming, further delaying the achievement of the results and the beginning of a targeted therapy. Moreover, clinical break points have only been defined for the main antifungal agents and for the most common species such as *Candida albicans*, *Candida glabrata*, *Candida tropicalis*, *Candida parapsilosis*, *Cryptococus* and *Aspergillus fumigatus*, *Aspergillus terreus*, *Aspergillus niger*, *Aspergillus flavus* and *Aspergillus nidulans*. In addition, clinical break points vary between EUCAST and CLSI, making the interpretation of the results difficult. Considering these difficulties, several methods based on the real-time polymerase chain reaction have been developed to identify the fungi directly from the sample and to evaluate the presence of mutations in genes associated with the antifungal resistance [117,120].

The development of AmB resistance is rare, probably because it interacts directly with ergosterol and not with an enzyme. However, it has been occasionally reported to be associated with *Candida* sp. and *Aspergillus* species. The resistance of *Candida* sp. and in particular of *Candida aurisi* to AmB is due to point mutations in genes that control ergosterol biosynthetic pathways such as ERG2, ERG3, ERG5 and ERG11 [121,122,123]. In addition, the occurrence of single nucleotide polymorphisms in an unnamed membrane transporter-encoding gene FLO8 has also been linked to AmB resistance in *Candida auris* [124,125]. A high rate of AmB resistance (approx. 50%) was also detected in *Trichosporon asahii*. Regarding *Cryptococcus* sp., resistance to AmB is rare [126]. Regarding the *Aspergillus* species, not only have mutations been identified in the *Candida* species, but they also have an intrinsic resistance mechanism among the isolates of *Aspergillus terreus* and *Aspergillus flavus* in comparison to other *Aspergilli*. This mechanism seems to be associated with the increase in catalase levels that counteracts the oxidative stress induced by AmB [22,115,123].

Regarding flucytosine, resistance mechanisms are associated with the occurrence of mutations in the FUR1 gene (uracil phosphoribosyl transferase) and in the FCY1 gene (cytosine deaminase). Mutations in these genes are associated with a decrease in the conversion of flucytosine into 5FU, the active metabolite. In addition, recent studies have also pointed out that defects in the DNA mismatch repair pathway of the fungal cell make the *Cryptococcus* species more susceptible to the occurrence of mutations and consequently more prone to developing resistance to flucytosine. Since the treatment of *Cryptococcus*-associated infection needs to be prolonged, it is associated with severe toxic effects and the resistance may develop easily; the use of flucytosine in monotherapy is not recommended. One of the possibilities is to use AmB, which at a low concentration maintains the ability to permeabilize the membrane, facilitating the entry of flucytosine. More recently, associations of flucytosine with triazoles have also been successfully used to treat difficult-to-treat infections related to the *Candida* species and also to *Cryptococcus* [127,128,129]. Resistance to the azoles seems to be associated with alterations in the genes involved in the ergosterol pathway and in the transport of the azoles into the fungi. One of the mechanisms is related to the cytochrome P450 enzyme lanosterol 14-α demethylase (CYP51). The binding of the azole to CYP51 inhibits the demethylation of ergosterol precursors and blocks ergosterol biosynthesis. The alterations in CYP51 may occur due to mutations in the cyp51A gene and/or due to the existence of tandem repeats (TRs) in its promoter region that lead to an increased expression of the gene [121,130,131,132].

The mutations induce amino acid substitutions that reduce the binding affinity of triazoles, and the TRs induce alterations in the enzyme structure, favoring the native substrate conversion and thus ergosterol biosynthesis, as evidenced in several strains of *Aspergillus fumigatus* [117,118,130,133]. Two of the most frequent combinations—mutations in the sterol demethylase and TRs—are the TR34/L98H and TR46/Y121F/T289A, which are associated with itraconazole and voriconazole resistance, respectively [117,121,122,131,132,133,134].

A second mechanism of resistance is associated with the overexpression of the efflux pumps. The ATP-binding cassette (ABC) superfamily and the major facilitator superfamily (MFS) are drug efflux pathways that cause the active efflux of azole drugs, thereby contributing to antifungal resistance. Previous studies evidenced that the exposure of *Aspergillus fumigatus* to voriconazole upregulates the CDR1B that encodes the transporter ABC11, contributing to azole resistance. Similar alterations were also observed in the *Candida* species. In addition, the overexpression of the MDR1 gene that encodes a transporter of the MFS has been associated with fluconazole resistance in *Candida albicans* [115,117,118,134,135].

Another mechanism of resistance in the *Candida* species is associated with point mutations in the ERG3 that encode the C-5 sterol desaturase enzyme. These mutations block the accumulation of 14-α-methyl-3,6-diol, the toxic sterol intermediate produced by the azoles in the absence of the mutations, allowing the survival of the fungi [121,122].

Due to the increased incidence of resistance to the azoles, recent studies are investigating other enzymes of the ergosterol synthesis pathway. One of these enzymes is the 3-hydroxy-3-methyl-glutaryl-coenzyme A reductase (HMG-CoA) protein, involved in the catalysis of the first step of ergosterol synthesis and encoded by the HMG1 gene in *Aspergillus fumigatus*. Mutations in HMG1 are associated with an accumulation of ergosterol precursors without altering CYP51 gene expression and an increase in the minimal inhibitory concentration (MIC) of itraconazole, voriconazole, posaconazole and isavuconazole. However, further studies are needed to better understand the mechanisms associated with these mutations [117,118,134].

The resistance to echinocandins is related to mutations in the FKS1 gene which encodes the catalytic subunit of the 1,3-β-D-glucan enzyme. This enzyme synthesizes 1,3-beta-glucan, a structural component of the fungal cell wall. Mutations in FKS1 decrease the IC50 of the enzyme by several orders of magnitude, elevate MIC values and result in cross-resistance to diverse echinocandins. In *Candida albicans*, the mutation in the serine 645 residue (S645) is associated with the most frequent resistance phenotype. In *Candida auris*, the most relevant mutation is in serine 639 residue (S369F). Regarding the FKS2 and FKS3 genes, less is known. Experimental studies have shown that the occurrence of deletions in these genes is associated with lower echinocandin susceptibility. In *Candida glabrata*, mutations occur in both FKS1 and FKS2. In *Aspergillus fumigatus*, the most relevant mutation associated with resistance is the substitution of S678P in FKS1 [41,91,133,136].

## 5. Development of New Antifungals

In recent decades, the number of invasive fungal infections has increased significantly. The increase in the number of immunocompromised patients due to malignancies, organ transplants, autoimmune diseases and the use of indwelling catheters and prosthetic devices were pointed out as being the main causes [16,28,29,78,137]. Considering these facts, the existence of effective antifungal drugs is a matter of survival. However, the available antifungal drugs have some drawbacks that limit the therapeutic success, such as occurrence of adverse effects associated with the necessary dose, with the prolonged treatment time or with the similarities between fungi cells and mammalian cells; difficulties reaching the site of infection; and the development of antifungal resistance [118,138].

Considering these limitations and in order to assist in the proper drug prescription, The Infectious Diseases Society of America (IDSA) and the European Society of Clinical Microbiology and Infectious Diseases (ESCMID) recommended that the use of echinocandins should be a first-line empirical treatment and primary therapy for suspected invasive candidiasis, and voriconazole should be a first-line therapy for invasive aspergillosis [139,140,141].

Having in mind the limited number of antifungal drugs and the development of resistance, there have been several attempts to use a combination of drugs, especially in patients diagnosed with invasive fungal infection who had failed to respond to antifungal monotherapy [19,26,138,142]. Since flucytosine inhibits the synthesis of DNA, polyenes bind to the membrane sterols impairing their function, azoles inhibit the synthesis of the cell membrane ergosterol and echinocandins inhibit the synthesis of β-(1,3)-D-glucan, a component of the cell wall. Several combinations, such as flucytosine with AmB, or caspofungin with voriconazole, or caspofungin with AmB, may hypothetically have a synergistic or additive effect. These combinations could improve the efficacy and the safety of the treatment [46,47,66,143].

In fact, the use of intravenous AmB combined with oral flucytosine was recommended by the guidelines for the prevention and treatment of opportunistic infections in adults and adolescents with HIV [26,129].

However, the analysis of the results obtained using monotherapy and combined therapy in patients from intensive care units was controversial, as evidenced in the retrospective analysis performed by Yang et al. [138]. According to this analysis, the combination of caspofungin with AmB and caspofungin with voriconazole did not result in a better outcome. Nevertheless, the mortality rate at 90 days was lower for caspofungin and voriconazole than for voriconazole in monotherapy. However, as Yang et al. emphasized, it is important to consider that there are significative differences in study design, and the number of patients in each treatment group was reduced [138].

To surpass all of these difficulties, it is important to develop new drugs. In recent years, several attempts have been made in order to develop new antifungal drugs [35,144,145,146,147,148].

Rezafungin is a newly (first approved on 22 March 2023) FDA-approved echinocandin. Its use is indicated in patients aged 18 years or older who have limited or no alternative options for the treatment of candidemia and invasive candidiasis. The efficacy of rezafungin was also evaluated in the ReSTORE study which was a multicenter, double-blind, double-dummy, randomized phase 3 trial conducted in 66 tertiary care centers in 15 countries that aimed to compare the efficacy and safety of intravenous rezafungin versus intravenous caspofungin in patients with candidaemia and invasive candidiasis. The study concluded that rezafungin was non-inferior to caspofungin for the primary endpoints of Day 14 global cure (EMA) and 30-day all-cause mortality (FDA). There were no concerning trends in treatment-emergent or serious adverse events. These phase 3 results showed the efficacy and safety of rezafungin and supported its ongoing development [90,147].

Ibrexafungerp is also a new orally available echinocandin indicated for the treatment of adult and postmenarchal pediatric females with vulvovaginal candidiasis. The in vitro studies pointed out that it is effective against most of the *Candida* species, including *Candida krusei* and *Candida auris*, it retains activity against most fluconazole-resistant *Candida* species and no resistance development was observed after monthly ibrexafungerp dosing in patients with recurrent vulvovaginal candidiasis [144,147].

Olorofim (formerly, F901318) is a fungicide drug from the orotomide group that targets the dihydroorotate dehydrogenase enzyme and inhibits pyrimidine biosynthesis and consequently the formation of UDP-sugars and substrates for 1,3-β-D-glucan synthase. Olorofim is being trialed in a phase 2b open-label study in patients who have limited treatment options for difficult-to-treat, invasive, azole-resistant aspergillosis, scedosporiosis and lomentosporiosis. In addition, there is a phase 3 trial (“OASIS”) that aims to compare olorofim versus AmBisome^®^ followed by the standard of care in patients with proven or probable invasive fungal infection due to the *Aspergillus* species (NCT05101187). The antifungal drug received orphan drug designation from the FDA and from the EMA for the treatment of cocidioidomycosis, scedosporiosis, invasive scopulariopsis and invasive aspergillosis [136,145,147,149].

Fosmanogepix is an N-phosphonooxymethyl prodrug that is metabolized by the mammalian systemic phosphatases to the active moiety manogepix (MGX; formerly APX001A). MGX targets the conserved fungal enzyme Gwt1, which catalyzes an early step in glycosylphosphatidylinositol (GPI)-anchored biosynthesis. GPI-anchored mannoproteins allow fungi to adhere to mucosal and epithelial surfaces. In addition, some fungal adhesins and virulence factors are also derived from GPI-anchored proteins [126,147,150]. In vitro activity has been reported against *Candida* sp., including *Candida albicans*, *Candida auris* and *Candida glabrata*, *Cryptococcus neoformans* and *Cryptococcus gatti*, *Coccidioides* sp., *Aspergillus* sp., including azole-resistant *Aspergillus fumigatus*, *Fusarium* sp., *Scedosporium* sp., *Lomentospora prolificans* and other rare molds. However, it has also been reported that MGX lacks in vitro activity against *Candida krusei* and some of the *Mucorales*, including variable activity against *Rhizopus* and *Lichtheimia*. The clinical trials evidenced that fosmanogepix is well tolerated and had reduced adverse effects. The FDA gave a fast-track status to fosmanogepix for invasive candidiasis, aspergillosis, scedosporiosis, fusariosis, mucormycosis, cryptococcosis and coccidioidomycosis [147,151].

Opelconazole (PC945) is an inhaled triazole with a mechanism of action similar to the other azoles. Upon inhalation, the main target is the lungs. However, due to alterations in the formulation, opelconazole seems to achieve high local concentrations, prolonged lung retention and low plasma concentrations, reducing the systemic adverse reactions and drug–drug interactions. Considering the in vitro studies, opelconazole is recommended for the treatment of pulmonary aspergillosis in non-neutropenic patients without disseminated infection. A phase 2 study is also evaluating the safety and tolerability of opelconazole in lung transplant recipients. On 13 January 2023, the European Union gave the designation of orphan medicine for the treatment of invasive aspergillosis to opelconazole [72,73,147,152,153].

Oteseconazole (VT-1161) is an oral tetrazole approved by the FDA in 26 April 2022. It also targets CYP51 and it was designed to have an improved safety and efficacy profile. In vitro, it has activity against *Candida albicans*, *Candida glabrata* and fluconazole-resistant *Candida albicans* from acute and recurrent vulvovaginal candidiasis. VIOLET, a phase 3 multicenter, randomized, double-blind, placebo-controlled trial was designed to evaluate oteseconazole efficacy in the treatment of recurrent vulvovaginal candidiasis up to Week 48, the time to first recurrence, safety and patient outcomes. The study concluded that oteseconazole was effective in preventing acute vulvovaginal candidiasis recurrence and treating recurrent vulvovaginal candidiasis in the CL-011 and CL-012 trials, with reduced adverse effects [73,154,155].

Quilseconazole (VT-1129) is an oral tetrazole with in vitro activity against the *Cryptococcus* species. It was also developed to have an improved safety and efficacy profile. FDA gave the orphan drug designation for the treatment of this life-threatening disease to quilseconazole and granted Qualified Infectious Disease Product designation to the treatment of *Cryptococcus*-associated infections [156,157].

VT-1598 is also a tetrazole that has been recognized by the FDA as being a Qualified Infectious Disease Product and been given fast-track status and orphan drug designation for the treatment of coccidioidomycosis. In vitro VT-1598 also presents activity against *Candida auris*, *Aspergillus* and *Rhizopus arrhizus.* VT-1598 has a formulation for oral administration in the form of tablets and a formulation for intravenous use. Results from clinical trials are needed to evaluate its efficacy [94,126].

These new drugs show that there is recognition of the need to improve the treatment of antifungal infections. One of the important aspects to highlight is the concern to design drugs with reduced adverse effects and also the exploration of new mechanisms of action in order to improve efficacy and reduce the development of resistance. Despite all of the efforts, some time will still be needed to be able to evaluate the clinical performance of these new drugs.

## 6. Conclusions

The increase in fungal infections is a current fact in clinical practice, which highlights the need for effective therapies. The in-depth knowledge of the available therapeutic options and of the adverse problems that may define narrow safety margins specific to each drug and patient emphasize the need for precision medicine and therapy.

In this review, we have highlighted the role of pharmacogenomics, pharmacokinetics and pharmacodynamics as crucial tools to achieve the best clinical practice in the use of antifungals, opening the door to defining efficacy algorithms and bringing together experts in clinical and laboratory areas, in the best interest of patient safety and treatment.

In addition to the pharmacological properties of the antifungal drugs, our review also highlights the need to know the epidemiology of fungal infections in order to be aware of the existence of antifungal resistance. The adequate knowledge of the epidemiology will allow for the establishment of an adequate therapeutic plan, avoiding the use of drugs for which the fungi are resistant.

In conclusion, this review addresses in an integrated and sequential manner the pharmacological characteristics of antifungals, the morphological and genetic characteristics of fungi that may contribute to the development of resistance and the mammalian host characteristics that may limit antifungal efficacy.

## Figures and Tables

**Table 1:** Mechanisms of action of the antifungal drugs.

Site of Action	Class of Drug	Examples	Mechanism of Action
**Loss of cell membrane integrity**	**Polyenes**	Amphotericin B deoxycholate, Liposomal amphotericin B, Nystatin, Natamycin	Binds to ergosterol, a specific steroid-alcohol of fungi. The polyene-ergosterol complex creates pores in the fungal cell membrane, leading to electrolyte leakage, cell lysis and cell death [39].
	**Azoles:**	Ketoconazole, miconazole, clotrimazole, itraconazole, isavuconazonium sulfate (isavuconazole), fluconazole, voriconazole, posaconazole	Non-competitive inhibitors of the fungal enzyme lanosterol 14-alpha-demethylase, a rate-limiting enzyme in the fungal biosynthetic pathway of ergosterol. This action destabilizes the fungal cell membrane, causing cell content leakage, lysis and cell death [31,37].
	**Allylamines**	Terbinafine	Inhibitor of the squalene epoxidase involved in the conversion of squalene to lanosterol, a precursor of ergosterol and cholesterol [40].
**Loss of cell wall integrity**	**Echinocandins**	Caspofungin, Micafungin, Anidulafungin	Inhibitor of 1,3-β-glucan synthase [41].
**Mitotic inhibitors**		Griseofulvin	Mitotic inhibitor that binds to polymerized fungal microtubules, inhibiting de-polymerization and leading to the failure of fungal cell replication [42].
**Pyrimidine** **antimetabolite**		Flucytosin	Inhibitor of nucleic acid synthesis [43].

**Table 2:** Pharmacokinetic profile of antifungal drugs.

	Standard Dose (mg/kg)	Bioavailability (%)	Protein Binding (%)	Metabolism (CYP)	Excretion (% Not Metabolized)	Vd (L/kg)	CL (mL/h/kg)	t/2 (h)	Tmax (h)	Renal Impairment	Hepatic Impairment
**Polyenes**:											
Amphotericin B (liposomal)	3–4 mg/kg per day (5 mg/kg for mucormycosis, or even 10 mg/kg for *Mucorales* infections of the CNS)	-	95–99	-	Renal (20–33); hepatic (40–43)	0.05–2.2	1–23	13–24	4	No dose adjustment; consider nephrotoxicity	No dose adjustment; consider hepatotoxicity
**Azoles**											
Fluconazole	Intravenous: loading dose 12 mg/kg once Maintenance dose 6 mg/kg once daily Oral: depends on clinical indication	90	11–12	3A4 (10%)	Renal (64–90)	0.6–0.8	15–24	27–37	0.5–1	Dose reduction (by 50% for GFR 11–50 mL/min)	No relevant hepatic metabolism; consider hepatotoxicity
Itraconazole	Loading dose 200 mg b.i.d. Maintenance dose 200 mg once daily—200 mg b.i.d	55; depends on the pH	99	3A4 (active metabolite-hydroxytroconazole)	Hepatic—54% in feces (3–35 in urine)	11	Dose-dependent	15–42	2.5	No dose reduction; enhanced dose during continuous renal replacement therapy	Consider dose reduction; TDM
Voriconazole	Intravenous: loading dose 6 mg/kg b.i.d. on Day 1 Maintenance dose 4 mg/kg b.i.d. Oral: loading dose 400 mg b.i.d. on Day 1 Maintenance dose 200 mg b.i.d.	90–96; affected by food	51–67	2C19/2C9/3A4	Hepatic (<2; more than 80% metabolite in urine)	4.6	100	6–12	1–2	Standard dose; consider SBECD accumulation during i.v. infusion	Mild to moderate: 50% dose reduction; TDM recommended
Posaconazole	Oral suspension: therapeutic dose 200 mg q.i.d. or 400 mg b.i.d Prophylaxis 200 mg t.i.d. Tablet formulation: loading dose 300 mg b.i.d. on Day 1 Maintenance dose 300 mg once daily Intravenous: loading dose 300 mg b.i.d. on Day 1; maintenance dose 300 mg once daily	Variable; affected by food and low pH	>98	Glucoronidation via UGT	Hepatic—77% in the stool	3.7–20	100–485	15–35	3–6.3	No dose adjustment; in intravenous formulation, avoid because of SBECD accumulation, when GFR < 50 mL/min	No dose adjustment
Isovuconazole	Intravenous: loading dose 200 mg t.i.d. on Day 1 and Day 2 Maintenance dose 200 mg once daily Oral: loading dose 200 mg t.i.d. on Day 1 and Day 2 Maintenance dose 200 mg once daily	>98; unaffected by pH or food	>99	3A4/3A5; Glucoronidation via UGT	Hepatic—46% in feces (45 in urine)	6.5	30–70	80–130	2	Standard dose	Mild to moderate: enhanced levels; no dose reduction recommended by the manufacturer
**Echinocandins**											
Anidulafungin	Loading dose 200 (*T*inf, 180 min), maintenance dose 100 (*T*inf, 90 min)	-	99	Spontaneous degradation in plasma	Hepatic (chemical hydrolysis) (10)	0.6	15	40–50	-	No dose adjustment	Slightly lowered concentrations; no dose adjustment recommended
Caspofungin	Loading dose 70, maintenance dose 50 (70 if body weight >80 kg)	-	92.4–96.5	Independent CYP	Renal (chemical hydrolysis) (1.4)	0.3–2	10	8	-	No dose adjustment	Enhanced exposure in moderate hepatic impairment; dose reduction
Micafungin	50 for prophylaxis, 100 for candidaemia, 150 for oesophageal candidiasis	-	>99	3A	Hepatic (chemical hydrolysis) (<1)	0.3	12	13–20	-	No dose adjustment	Slightly lowered concentrations; contra-indicated in European SmPC
**Flucytosine**	25–37.5 mg/kg 4 times per day	90	3–4	Minimum	Renal (>99)	0.4–0.8	-	3–6	-	Dose reduction guided by glomerular filtration rate	Flucytosine should be avoided because of hepatotoxicity; no effect on pharmacokinetics because of renal elimination

The PK profile of each of these groups has well-defined characteristics that vary depending on their molecular weight, solubility, binding to plasma proteins and the genetic polymorphisms of CYP2C19 and CYP3A4 that affect their metabolization profile [44,45,46]. Vd—volume of distribution; CL—clearance; t1/2—half-life, refers to the time required for plasma concentration of a drug to decrease by 50%; Tmax—time to reach the maximum plasma concentration; SBECD—sulfobutylether-beta-cyclodextrin; CYP—Cytochrome; UGT—UDP-glucuronosyltransferases; TDM—therapeutic drug monitoring.

**Table 3:** PK/PD characteristics of antifungal drugs and recommendations for TDM.

	PK/PD Features	Recommendation for TDM	TDM Protocol	Comments
**Polyene**: Amphothecin B in lipid/liposomal formulations	Cmax/MIC is the best clinical response rate Cmax/MIC >= 4.5 is the required liposomal efficacy Cmax 14–29 mg/L (liposomal)	Not routinely, despite high intra- and individual intervariability, but for now no correlation between concentrations and efficacy/safety Lack of an objective therapeutic range	Amphotericin B blood measurements do not represent true liposomal exposure due to liposomal structure and plasma protein binding	Not recommended by experts, but TDM may be recommended for toxicity monitoring and therapeutic optimization
**Triazoles:**				
Fluconazole	AUC0-24 AUC/MIC or dose/MIC = 50–100 Cmax 20–20 mg/L Cmin 10–15 mg/L	Not routinely, but when the patient has renal replacement therapy, central nervous system (CNS) infection or when treating a microorganism with elevated MIC TDM is limited as AUC/MIC is not a clinically practical monitoring parameter	As the best form of prediction is the AUC, collections should be made at least 2 h after the dose and be valid 30 min before the next administration	Not recommended by experts, but TDM can be useful for pediatrics or when using renal replacement therapy
Isavuconazole	AUC0-24/MIC correlated with efficacy Cmax 4.4 mg/L	Not routinely, despite being indicated in cases of therapeutic failure (as with fungi with high MICs ≥ 2 mcg/mL, such as with *Fusarium* sp.), obesity, non-adherence, drug interactions that reduce isavuconazole concentrations and age below 18 years	Linear kinetics for doses > 600 mg/day	Not recommended by experts; more studies are needed
Itraconazole	Cmin > 1 mg/L for efficacy and <5 mg/L to minimize toxicity Cmin > 0.5 mg/L for prophylaxis Bioassay assessments can be 3–7 times higher than HPLC	Indication for TDM is strongly recommended Mainly for evaluation of oral absorption	Non-linear kinetics; slow accumulation with ineffective half-life Steady state 7 to 15 days Cmin harvest	Calculation of the itro/hydroxytroconazole ratio to characterize the patient’s metabolic phenotype
Voriconazole	Cmin > 1–1.5 mg/L for treatment efficacy Cmin < 5–6 mg/L to minimize toxicity Cmin/MIC = 2–5 (MIC estimated by CLSI)	Indication for TDM is strongly recommended Should be dosed for switches from IV to oral or in unstable patients	Non-linear kinetics; accumulation via elimination saturation Steady state 2 to 5 days Cmin harvest	Calculation of N-oxidoVori/Voriconazole ratio to characterize the patient’s metabolic phenotype
Posaconazole	Cmin > 0.5–0.7 mg/L in SS for treatment efficacy (prophylaxis) Cmin > 1 mg/L (treatment)	Indication for TDM is strongly recommended, especially when used orally and for invasive infections	Steady state 5 to 7 days Cmin harvest	It is recommended by experts
**Echinocandins**	Cmax/MIC and AUC0-24/MIC is the best clinical response rate Cmin = 1 mg/L for invasive infections	The indication for TDM is unclear However, it should be considered for patients with highly variable factors		Not recommended by experts, but may be considered in newborns, children, adolescents, obese patients, patients with renal failure and critical and hematological patients
**Flucytosine**	Cmax 30–80 mg/L is the best clinical response rate (cryptococci mostly) Cmax >100 mg/L should be avoided—toxicity Cmin < 20–40 associated with resistance	Indication for TDM is strongly recommended	Steady state 3 to 5 days Cmax—obtain 2 h after oral dose or 30 min after iv dose	It is recommended by experts

AUC—area under the curve; Cmax—maximum plasma concentration; MIC—minimum inhibitory concentration; Cmin—minimum plasma concentration.

## Data Availability

Not applicable.
